# Insights into Characterization Methods and Biomedical Applications of Nanoparticle–Protein Corona

**DOI:** 10.3390/ma13143093

**Published:** 2020-07-10

**Authors:** Yan Li, Jae-Seung Lee

**Affiliations:** Department of Materials Science and Engineering, Korea University 145 Anam-ro, Seongbuk-gu, Seoul 02841, Korea; liyn18@korea.ac.kr

**Keywords:** nanoparticles, protein corona, characterization, biomedical application

## Abstract

Nanoparticles (NPs) exposed to a biological milieu will strongly interact with proteins, forming “coronas” on the surfaces of the NPs. The protein coronas (PCs) affect the properties of the NPs and provide a new biological identity to the particles in the biological environment. The characterization of NP-PC complexes has attracted enormous research attention, owing to the crucial effects of the properties of an NP-PC on its interactions with living systems, as well as the diverse applications of NP-PC complexes. The analysis of NP-PC complexes without a well-considered approach will inevitably lead to misunderstandings and inappropriate applications of NPs. This review introduces methods for the characterization of NP-PC complexes and investigates their recent applications in biomedicine. Furthermore, the review evaluates these characterization methods based on comprehensive critical views and provides future perspectives regarding the applications of NP-PC complexes.

## 1. Introduction

Advances in material science have made it possible to synthesize nanoparticles (NPs) with specific sizes, shapes, compositions, and surface properties; these attributes play decisive roles in the functions of NPs. Recently, modifying the surfaces of nanomaterials has led to significant developments in applications in biomedicine, therapeutics, and diagnostics [1,2,3,4,5]. Although there are a large number of studies involving NPs in the literature, few of these studies are applied in clinical trials. There is a lack of clear approaches for synthesizing NPs to be used in vivo. Moreover, there is a wide gap between the theoretical studies and clinical applications, owing to limited understanding regarding NPs in the physiological milieu. Thus, it is essential to comprehensively elucidate the properties of NPs in physiological systems to extend their biomedical applications.

When nanomaterials are exposed to physiological milieus as contrast agents and drug carriers, the surfaces of the NPs are adsorbed by multiple proteins, forming a complex called NP–protein corona (NP-PC). The adsorption of the proteins has effects on the physicochemical properties of the NPs, including the sizes, shapes, compositions, and surface charges [6,7,8,9,10,11], providing the NPs with biological identities that are different from those of the bare NPs (NPs without PC coating but maybe with some organic ligands on them) [12,13,14,15,16]. Meanwhile, the adsorption of proteins on an NP’s surface can also cause the proteins to undergo conformational changes, which may further affect the biofunctionality of the NPs, including cell recognition and cell signal conductivity [17,18,19]. Focusing on bare NPs does not help for understanding their appropriate applications in biomedicine, and there are growing concerns regarding the interactions between NPs and proteins at the intra- and extra-cellular levels, as these proteins play an important role in the pharmacokinetics and biological performance of the NPs [20,21,22,23,24,25].

**Figure 1:**
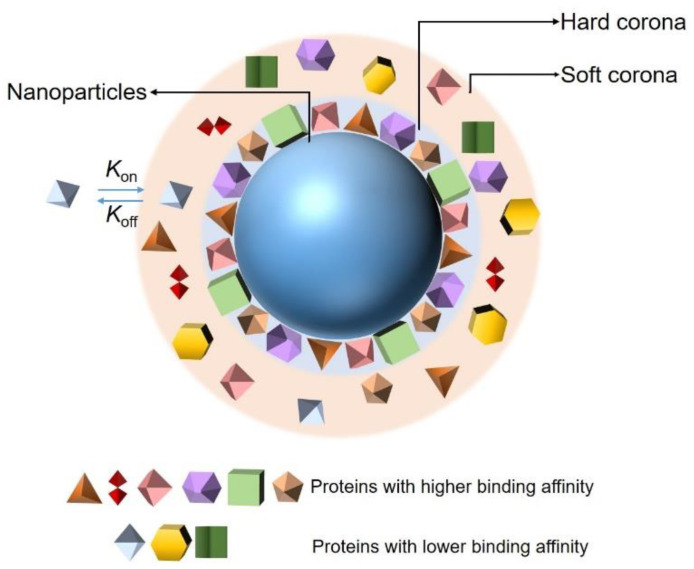
Schematic representation of the current protein corona hypothesis.

The adsorption of proteins on the surfaces of NPs is controlled by protein–NP interactions and protein–protein interactions. There are more than 3700 proteins in the plasma, and the adsorption of those proteins at any given time is determined by their concentrations and the equilibrium binding constant of each protein to the NPs. In addition, the composition of a PC depends on the size, shape, surface charge, and surface composition of the NP. Although the composition of a PC is controlled by multiple factors, in general, according to the binding affinity and rate of exchange of proteins from the NP’s surface, the PC around an NP can be divided into two parts: a hard corona and soft corona (Figure 1) [26,27,28]. Proteins that have been proven to have a high binding affinity with the surfaces of NPs belong to hard coronas and are generally located in the inner layer of the PC. Their rate of exchange from the NP surface is slow, but the time is enough for the internalization of the NPs by cells [29], inducing an irreversible binding between the proteins and NPs. By contrast, the association of proteins constituting soft coronas with NPs is less stable than that of those of hard PCs [30]. They are loosely attached to the NPs’ surface and relocate from the NP’s surface rapidly. Furthermore, they may interact with the formed hard PC layer on the NP’s surface, via weak protein–protein interaction. They are distributed in the outer layer of the PC and easily exchanged with the proteins existing in the biological environment, owing to the abundance of proteins in biological fluids and their direct contact with each other [31]. In addition, the adsorption of proteins on the surfaces of NPs is a dynamic process [32,33]. It is associated with the continuous adsorption/desorption equilibrium of the proteins on and off the NP surfaces; the kinetics are described by association (*k*_on_) and dissociation (*k*_off_) constants. The values of *k*_on_ and *k*_off_ are related to the contact frequency and binding energy of the proteins and NPs, respectively. The balance between these two factors plays an important role in determining the affinity of a protein to an NP and is generally defined as the dissociation constant (*K*_d_). Proteins in plasma exhibit diverse affinities to different NPs, resulting in specific PC forms depending on the size, surface charge, and morphology of the given NP. Finally, the PC acts as an identifier of a specific NP in a physiological environment and allows the NP to be detected by cells, thus enabling the cells to interact with the NP as well. Therefore, an accurate and extensive characterization of NP–PC interactions will help in exploring the potential uses of NPs in biological applications and will contribute to extending the understanding of the toxic effects of NPs. Meanwhile, it will be conducive to reducing the toxicity of NPs to the human body.

This review introduces approaches for characterizing NP-PC complexes, including the characterization of the binding affinity, the location of binding sites, identifying the compositions of PCs, and distinguishing conformational changes in PCs. Critical issues regarding the approaches are presented, according to their features. Furthermore, we overview the recent applications of NP-PC complexes in biomedicine. It is anticipated that this review could lead to more practical and meaningful guidelines for the biomedical applications of NP-PC complexes.

## 2. Characterization Approaches

Developments in nanotechnology have led to increasing applications for nanomaterials with various sizes, compositions, and morphologies. The PC—which transforms many of the physicochemical properties of NPs such as the size, surface charge, and surface composition—greatly affects the functionality and interactions of NPs with biological systems and plays a crucial role in the availability of NPs in biomedicine and therapeutics. An increasing number of studies have concentrated on the characterization of NP-PC complexes. Employing appropriate approaches to characterize NP-PC complexes accurately is indispensable for extending the use of NPs in therapeutics and nanomedicines.

### 2.1. Nanoparticle–Protein Corona (NP-PC) Size, Shape, and Surface Charge

The physio-chemical characterization of the NP-PC complex is of the highest importance for understanding the impacts of NPs in biological media. A comparison of the sizes, shapes, and zeta potentials of NPs in the presence and absence of PCs provides information regarding the thickness of coronas and PC-mediated surface charge changes in the NPs.

**Figure 2:**
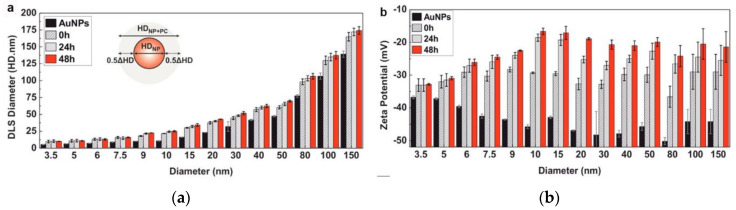
Dynamic light scattering (DLS)-measured hydrophobic diameter (HD) (**a**) and zeta potential (**b**) of purified Au nanoparticles (NPs) of different sizes after their exposure to cCCM for different times. Reprinted with permission from [10]. Copyright (2017) American Chemical Society.

Dynamic light scattering (DLS) is the most conventional method for determining the hydrodynamic size distribution of the NPs before and after PC adsorption, based on their Brownian motion [34,35]. DLS measures the time-dependent scattering intensity fluctuations caused by Brownian motion and uses the Stokes–Einstein equation to relate the diffusion coefficient to the NP size [36]. DLS is very sensitive towards soft flexible biological molecules and is therefore suitable for investigating protein coating-induced size changes in NPs. Several studies using DLS reveal an increase in the NP diameter after PC formation around an NP [10,35,37]; for example, the sizes of AuNPs have been reported to increase when incubating in complete cell culture media (cCCM) (Figure 2a) [10]. Although DLS is a powerful, fast, and nonperturbative method for characterizing NP dispersion, its resolution is limited. It generates a scattering signal of the entire dynamic range of NP sizes in one sample and analyzes them simultaneously; NPs with similar sizes cannot be distinguished, and slight shifts in size before and after protein adsorption cannot be detected. When introducing DLS to a particle mixture with a broad size distribution, smaller sizes may be ignored. In addition, samples for DLS measurements not only need to be monodispersed but should also be dust free, thus further limiting their application. Many DLS systems are equipped with electrophoretic light scattering and allow zeta potential measurements. The zeta potential characterizes the net charge of a particle surface in a given dispersant. It can be used to quantitatively measure the coating of proteins on an NP’s surface, as there will be a concomitant change in the zeta potential with the growing protein coating. Most NPs are negatively charged after PC adsorption at a physiological pH [38,39,40]; as shown in Figure 2b, the zeta potentials of purified AuNPs of different sizes increase but remain negatively charged when incubating in the cCCM [10]; changes in the NP surface charge may result in a drop in the electrostatic stability but an increase in the steric stability of the system [37]. The above alterations make the zeta potential a key indicator for investigating PC adsorption on the surfaces of NPs.

Another common approach for analyzing the sizes of NPs is differential centrifugal sedimentation (DCS). DCS is capable of separating the components of a mixture based on their densities and sizes, as larger and denser components require lower centrifugal forces to sediment. It is capable of analyzing the sizes of particles in the range of 0.01–40 μm and has been widely applied in measuring the sizes of NP-PC complexes [21,41]. For NPs with homogenous density and a simple shape, the particle size can be obtained directly, via the time taken by the NPs to travel from the center of a DCS disk through a defined viscous sucrose gradient under a strong centrifugal force to a detector placed at the outer rim of the disk [26]. Particles with an inhomogeneous density or irregular shape can also be distinguished by the different arrival times. Compared to DLS, a significant advantage of a DCS measurement is that it allows for the size analysis of particles with a broad size range within one sample [21,26,42]. Moreover, the measurements of particles can be performed in any possible medium, even in the presence of biomolecules, owing to their distinguished sedimentation times. It allows us the investigation of the properties of NP-PC complexes, even in vivo. However, it is notable that this technology may risk exposing the samples to contamination and may exhibit poor recovery.

**Figure 3:**
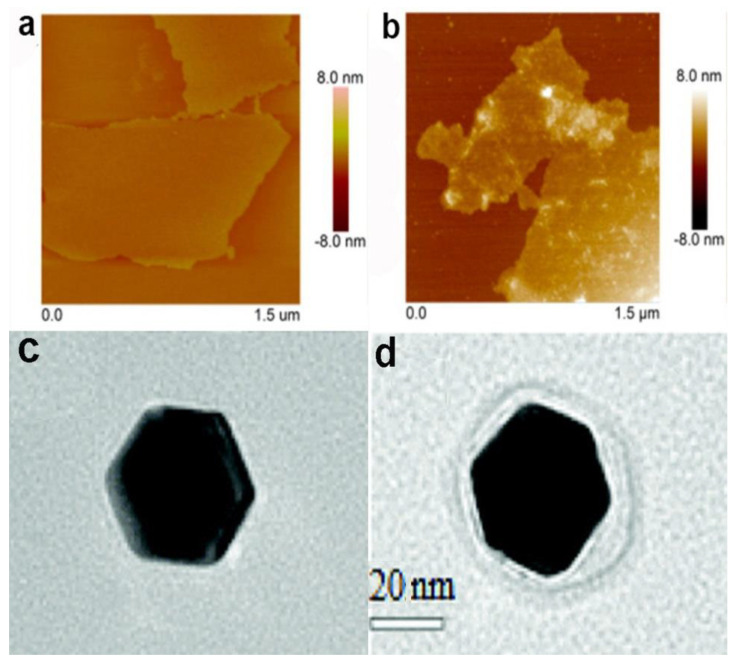
Atomic force microscopy (AFM) images of bare-graphene oxide (**a**) and graphene oxide–bovine serum albumin (BSA) complex (**b**). Reprinted with permission from [52]. Copyright (2015) American Chemical Society. TEM images of a single 40 nm AuNP (**c**) and 40 nm AuNP–human serum albumin (HSA) complex (**d**). Reproduced from [48] with permission from The Royal Society of Chemistry.

Particle sizes measured using DLS may be influenced by particle agglomeration. Therefore, as shown in Figure 3, transmission electron microscopy (TEM) or atomic force microscopy (AFM) images of NPs and NP-PC complexes are often obtained to determine whether the NPs have aggregated before DLS measurements [43,44,45,46,47]. Meanwhile, TEM and AFM can be used to evaluate the sizes of particles and the thickness of PCs. The size characterizations from DLS and TEM (or AFM) are helpful in eliminating ambiguities regarding the sizes of the NPs or the NPs’ agglomeration(s). The advantage of TEM or AFM with respect to DLS is that they provide clear and intuitive information regarding NPs’ sizes and shapes and the coating of the PC around the NP’s surface [48,49,50]. However, the drying and staining during the sample preparation process for TEM may affect the morphology of the NP-PC complexes [26,51]. The sample preparation for AFM is easier than that for TEM, as sample labeling is not required. However, the lengthy operation time remains a significant limitation of AFM and can lead to thermal drift in the sample.

As a powerful size analyzer, small-angle X-ray scattering (SAXS) has recently gained increased attention for the characterization of NPs and NP-PC complexes. Particles with the sizes in the range of 1–100 nm, especially ranging from 5 to 25 nm, can be measured using SAXS [53]. Furthermore, it provides information on the shapes of NPs and NP-PC complexes, evolutions of interactions, and morphologies of the resultant structures in NP–protein systems [54,55]. Compared with DLS, SAXS provides extensive structural information regarding NP-PC complexes such as the size and shape of NPs and NP-PC complexes, and studies regarding particles with larger size ranges. It also provides insights into the structure and thermodynamics of NP–protein dissociation under physiological conditions. Smaller samples are required for SAXS than DLS; however, the instrument for SAXS is very large and costly.

As mentioned above, DLS is challenging for measurements of polydisperse particles. As an alternative, a technique called NP tracking analysis (NTA) can accurately measure the appearance of polydispersity in a sample [56]. An NTA device is equipped with a laser light scattering microscope and a charge-coupled device camera, allowing for the visualization and recording of NPs in solution. Coating proteins on the NP surfaces increases the mass of the NPs and slows Brownian motion. NTA can track the Brownian motion of many particles simultaneously and can be used to measure a number-average particle size ranging from 30 nm to 1000 nm, based on diffusion coefficients [57]. The lower limit of the approach can vary depending upon the refractive index of the particle. It confers a better capability for quantifying polydisperse samples than DLS. In addition, as NTA tracks the Brownian motion of each particle, it can provide information on the particle concentration and size more accurately and rapidly than TEM and AFM. It has also been proven to be able to detect protein aggregation [58], which is difficult for other approaches to achieve.

### 2.2. Binding of Proteins to NPs

Generally, proteins with different binding energies exhibit different affinities to NPs during the formation of coronas. According to the binding capacity, PCs are divided into hard coronas and soft coronas. Different types of coronas confer diverse biological identities on NPs; therefore, the characterization of the binding between proteins and NPs plays a central role in the evaluation of NPs’ applications.

**Figure 4:**
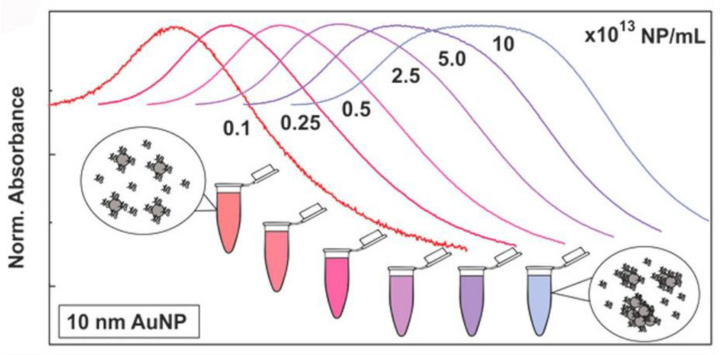
Normalized UV-vis spectra of AuNPs in cCCM at different concentrations. The changes in the LSPR band indicate that AuNPs aggregated in the cCCM when exposed to the medium at high concentration while their stability was not compromised at low concentration. Reprinted with permission from [10]. Copyright (2017) American Chemical Society.

UV-visible (UV-vis) spectroscopy derives data from the transition of electrons in metallic NPs; this process is highly sensitive to the surroundings of the NPs at the molecular level. Metallic NPs (typically gold NPs (AuNPs) and silver NPs (AgNPs)) exhibit a characteristic absorbance peak in the visible range, called the surface plasmon resonance (SPR) band [59,60]. UV-vis spectrometry can be employed to monitor NP–protein interactions. With protein adsorption, the characteristic peak usually undergoes a red shift owing to the size and surface changes (Figure 4), which are related to the collective oscillation of the surface electrons present on metal NPs [10]. It is a fast, easy, and applicable approach for measuring PC-induced changes in the dielectric environment immediately surrounding NPs [61]. However, the UV-vis absorption spectra of NPs are strongly affected by external environmental factors including the pH, temperature, and solvent, and complementary methods are required for the further characterization of the protein–NP complex. In addition, as the SPR phenomenon is only present in plasmonic NPs, UV-vis spectroscopy cannot be used in non-metallic systems.

**Figure 5:**
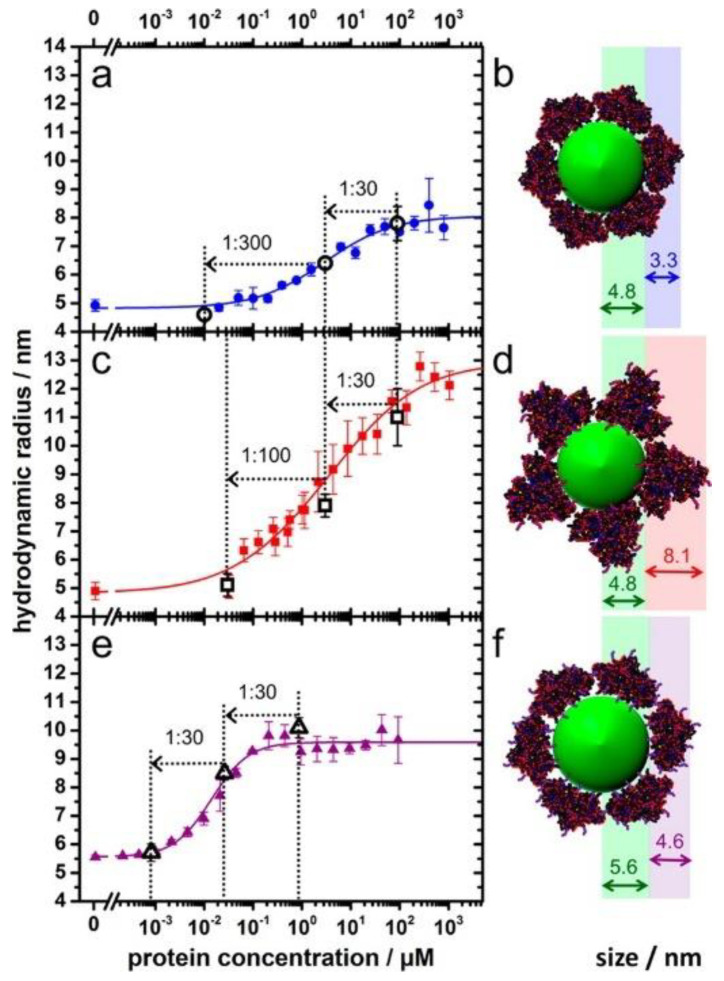
Adsorption of HSA (**a**,**b**) and succinic anhydride (**c**,**d**) and ethylenediamine surface (**e**,**f**) modified HSA onto dihydrolipoic acid-coated quantum dots obtained from fluorescence correlation spectroscopy (FCS). Reprinted with permission from [65]. Copyright (2014) American Chemical Society.

Owing to the associations of NPs with proteins, the sizes of the NPs usually increase, resulting in increased diffusion times. When using fluorescently labelled NPs or proteins, changes in the diffusion time can be detected by fluorescence correlation spectroscopy (FCS), and information regarding the tendency of the protein binding can be generated. FCS allows for measurements regarding the binding kinetics and thermodynamic properties of proteins as related to NPs [62,63]. Moreover, it is a powerful technique for investigating corona formation, along with the stability and aggregation states of the NPs [64]. As shown in Figure 5 [65], succinic anhydride modified human serum albumin (HSA) forms a thick PC of 8.1 nm and presents decreasing binding affinity to the quantum dots. By contrast, HSA and ethylenediamine surface modified HSA show enhanced binding affinities to the quantum dots, by forming PCs of 3.3 nm and 4.6 nm around the dots, respectively. Notably, FCS can be conducted inside cells and can elucidate the influence of the intracellular nanomaterial translocation on the PCs. Herein, plasmonic NPs are not needed; nevertheless, small amounts and low concentrations of fluorescents are required. The samples or cells are not destroyed during the measurements; however, the addition of fluorescents may alter the NP-PC interactions. It is important to choose the appropriate fluorophore for FCS experiments. In addition, the inner filter effect and light scattering from proteins or NPs may complicate the interpretation of fluorescence experiments. Thus, appropriate control experiments and a proper model for data fitting are required to eliminate any interferences from the environment.

Quartz crystal microbalancing (QCM) is an underexploited approach for characterizing the binding between NPs and proteins. In general, with increasing PC adsorption, the masses of NPs increase, and hence, QCM can potentially describe the quantitative binding profiles of the NP-PC interactions [66,67]. QCM measurements are usually conducted by immobilizing proteins and NPs on a gold surface. The gold surface is located on a quartz crystal and is placed between two electrodes. Real-time and quantitative NP-PC interactions are obtained by measuring the mass changes using sensors. The association and dissociation constants are analyzed based on the Langmuir adsorption isotherm. It is sensitive enough to detect monolayer protein formation around NPs but is applicable only to aggregate formation and not solutions.

Isothermal titration calorimetry (ITC) is a typical technique for providing thermodynamic information, such as the stoichiometry, enthalpy, entropy of binding, binding free energy, and association constants regarding the interactions between small molecules (NPs) and biomolecules (proteins), using a simple titration experiment [18,48,68,69]. In general, proteins are gradually injected into an NP solution in a sample cell, and the time-dependent heat changes in the sample and reference cells during the binding process are recorded. Thermodynamic components (such as the enthalpy and entropy of binding) can be calculated with the help of isothermal functions. When the concentrations of the NPs and proteins are known, the number of coated protein molecules per NP and binding affinity can be obtained. ITC is a straightforward approach for characterizing the capacity of proteins to bind to NPs, without separation and isolation processes. One of the challenges in studying NP–PC binding is the accuracy of the obtained thermodynamic components, which may be influenced by many factors during the fitting process. It is also challenging to interpret the binding between NPs and proteins when the binding process does not produce detectable heat changes. If the adsorption process involves multiple binding and unfolding steps, the heat changes related to NP–PC binding are hard to recognize. However, ITC remains an applicable technique for investigating the capacity of proteins to bind to NPs.

Nuclear magnetic resonance (NMR) is known to provide detailed information regarding the structures, thermodynamics, and dynamics of molecules. NMR allows the characterization of the behaviors of PCs adsorbed around NPs, owing to its ability in probing the individual residues of proteins. It can be used to locate the binding site, which is difficult to obtain with other approaches. It is capable of tracking the chemical environmental changes of the coupled H and N atoms of proteins during the adsorption of proteins on NPs. Therefore, information on the conformational changes and binding sites can be obtained in the three-dimensional structure of the proteins [70,71,72]. It is a non-destructive technique and can be applied to a wide variety of samples in solution and in solid-state, providing direct and indirect measurements of molecular dynamics. By combining NMR with other analytical approaches, very detailed information regarding NP-PC binding can be obtained. The main limitation of NMR’s application is the ambiguous influence of the NP surface on the protein adsorption behavior. Current NMR approaches fail to detect signals for surface-bound proteins. With further developments in NMR techniques, more applications of NMR regarding NP–PC binding are expected.

**Figure 6:**
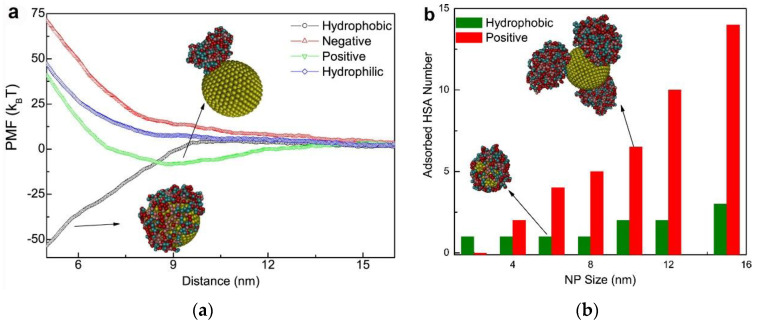
(**a**) Potential of mean force (PMF) of adsorption as a function of the distance (Z) from the center mass of the NP to that of HSA where the NP size is 10 nm. Effect of NP surface properties on protein adsorption. (**b**) The adsorbed HSA number as a function of the (hydrophobic and positively charged) NP sizes. Reprinted with permission from [73]. Copyright (2014) Elsevier Ltd.

In addition to experimental techniques, an increasing number of computer simulations have been applied in studies regarding NP-PC interactions [52,62,73,74,75]. Based on the experimental results, simulation studies are developed for explaining and predicting the PC adsorption around NPs. As shown in Figure 6 [73], steered molecular dynamics provides information about the potential of mean force (PMF) of NP-PC interactions as a function of the distance between NPs and HSA, as well as the adsorbed HSA number as a function of the NP sizes. It indicates that the adsorption of protein on an NP surface is dependent on its surface properties, and HSA can only adsorb onto charged nanoparticles and hydrophobic ones to form PCs. Computational studies provide information on protein orientation and conformation, with high spatial and temporal resolution. The binding of proteins to NPs is displayed as a function of the surface ligand structure, surface curvature, and protein identity. It is a powerful bioinformatics modeling technique for elucidating how a protein interacts with NPs. However, it is challenging to design appropriate parameters for characterizing the most important chemical and physical features of protein–NP systems.

### 2.3. PC Composition

The differences in the NPs’ properties and environments influence the PCs in terms of the interaction with the diverse protein compositions via adsorption. The compositions of PCs are complex and are unique to each NP. Identifying the composition of a PC is crucial for understanding the properties of NP-PC complexes. Generally, the identities of the proteins composing the coronas around NPs can be determined using gel electrophoresis, mass spectrometry (MS), and gel filtration chromatography.

Gel electrophoresis, which includes one-dimensional (1-DE) and two-dimensional (2-DE) gel electrophoresis, is the most commonly used approach for separating NP-PC complexes [76,77,78,79]. Approaches based on 1-DE or sodium dodecyl sulfate polyacrylamide gel electrophoresis (SDS-PAGE) separate proteins by molecular weight, after exposing samples to an electric field. Even though SDS-PAGE is a cheap, fast, and effective approach for separating and identifying the composition of a PC, it is relatively weak in the context of separating similar-size proteins within protein mixtures, as they have similar migration rates and may comigrate in the gel [80]. In terms of 2-DE, it separates proteins in two steps, i.e., isoelectric focusing and SDS-PAGE, in which proteins are separated depending on their isoelectric points and molecular weights, respectively. Then, the separated proteins are stained and analyzed via comparison with a 2-DE master map of proteins [81,82,83]. The 2-DE technique is useful for protein identification; however, it is time-consuming and is difficult to use for tracking the kinetics of NP–PC adsorption. As it provides qualitative and semiquantitative information, gel electrophoresis analysis is often followed by mass spectrometry analysis, to determine the identities of the separated proteins.

MS has been widely used for identifying the PCs (up to 100 kDa) around NPs, owing to its suitability for a range of NPs. Combining MS with chromatographic and gel-based approaches allows for the determination of the protein pattern of the entire PC around NPs [31,84,85]. Notably, the application of the digested proteins provides information on the proteins belonging to the hard PC that are closest to the NP surface. Even though MS is a destructive approach, it is a sensitive and efficient analytical technique for identifying PC compositions qualitatively and quantitatively. In addition, MS can be used to investigate bite sites of NP-PC complexes when combined with other techniques, such as hydrogen–deuterium exchange combined with proteolysis–MS or a chemical modification combined with proteolysis–MS [86,87].

Gel filtration chromatography is an effective approach for separating NP-PC complexes, isolating proteins from NP surfaces, and determining kinetic exchange rates for adsorbed proteins [7,88,89]. In gel filtration chromatography, proteins are separated according to their sizes, and exchange rates are determined by comparing the bound and free protein elution profiles [90,91]. It is assumed that all molecules have similar shapes, and the access of molecules into pores is proportional to their sizes. NPs with larger sizes are unable to enter the pores of the gel filtration media and emerge from the packed column early, whereas free proteins with smaller sizes are able to enter the pores and are separated based on their molecular weight. This approach is helpful in identifying the protein compositions of PCs and even in estimating the extent of protein aggregation. However, it is notable that if the NP-PC binding is weak, the binding equilibrium may be disturbed by the large dilution.

### 2.4. Conformational Changes in Proteins

Upon forming a PC on the surface of an NP, the conformation of the proteins involved in the PC may change, resulting in the alteration of the nature of the exposed epitopes in cells and a modification of the immune response. Various techniques have been applied to observe the conformational changes of proteins [92]; conventional approaches used for NP-PC complexes include circular dichroism (CD) spectroscopy, surface-enhanced Raman spectroscopy (SERS), and Fourier-transform infrared spectroscopy (FTIR).

**Figure 7:**
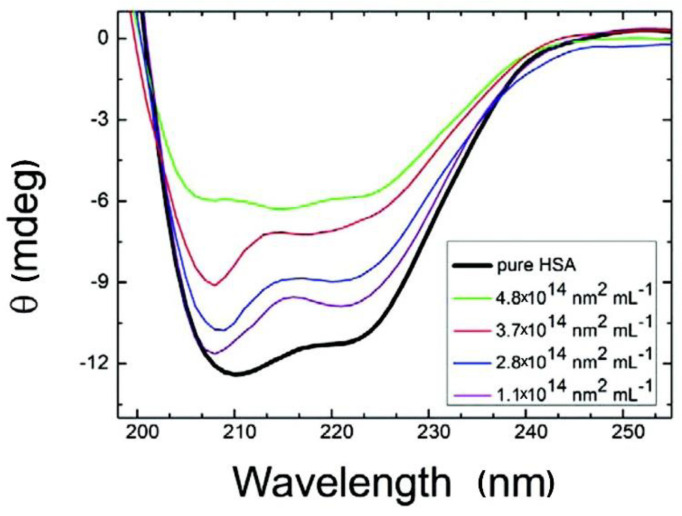
Circular dichroism (CD) spectra of HSA with citrate-stabilized silver NPs (diameter, 36 nm, with NP surface site concentrations ranging from 1.1 × 10^14^ to 4.8 × 10^14^ nm^2^·mL^−1^). Reprinted with permission from [99]. Copyright (2012) American Chemical Society.

CD has been extensively applied to the characterization of NP-PC interactions, owing to its sensitivity in detecting the NP interaction-induced conformational changes of secondary structures and the folding and binding properties of proteins according to chiral properties [93,94,95]. Each of the secondary structural elements—e.g., α-helices, β-sheets, and random coils—presents characteristic CD spectral features, which may be altered by binding with NPs. By comparing the characteristic CD spectral features before and after NP binding, the conformational changes of the PCs around NPs can be evaluated [96,97,98]. As shown in Figure 7 [99], losses in the α-helices of HSA as the concentrations of citrate-stabilized silver NPs increase are monitored using CD spectra. The chiral property is necessary for exhibiting a CD signal; however, an NP is not a chiral compound, and thus, NPs show no interference in the signal and/or interpretation of the data. It is a simple, low-sample-size, and less-time-consuming approach for monitoring conformational changes, and for estimating the secondary-structure composition of proteins with NP adsorption. The main limitations of this approach are that it is infeasible for the investigation of complex protein mixtures and that it cannot determine individual structural residues.

Raman spectroscopy (RS) is a scattering-based technique for measuring the NP conjugate in an aqueous solution with high spectral resolution [100]. It measures the molecular vibrations triggered by the inelastic scattering of light for protein conformation analysis. RS is a non-destructive, label-free, and highly specific technique for determining PC conformation but has limited application, owing to its low sensitivity and signal intensity. SERS has been applied to improve the signal intensity and selectivity of RS by using the localized surface plasmon resonance of noble metal NP substrates [101,102]. One drawback of SERS is that the possible alteration of the PC structure under intense laser heating may result in misleading results. In addition, the noble metal NPs, including AgNPs, AuNPs, and alkali metal NPs, as well as the SERS signal, are unstable in the presence of aggregation, which may limit the applications of SERS. Generally, SERS is not used independently but is combined with other approaches such as FTIR to identify the conformation of PCs around NPs. Similarly to CD spectroscopy, FTIR provides information on the conformational changes of proteins resulting from NP binding. The infrared activity of a molecule depends on the change of the electric dipole moment of the molecule upon the absorption of light. The absorption of infrared light when a molecule is excited from the ground vibrational energy level to a higher energy level provides information on molecular structures and molecular interactions. Therefore, FTIR is used to probe the absorption of infrared light on NP surfaces and to provide information on protein adsorption, including the evaluation of protein coatings on the surfaces of NPs and identification of a protein’s secondary structure [57,76,103,104]. The ability of SERS to measure NP-PC complexes in an aqueous solution is better than that of FTIR, and the spectra of SERS are simpler than those of FTIR; however, FTIR continues to be the most widely used technique for monitoring the conformation of PCs around NPs. Combining SERS with FTIR provides information regarding the conformation of the PC, in addition to vibrational and rotational parameters.

In addition to the above approaches, differential scanning calorimetry (DSC) can be used to detect conformational changes in PCs resulting from NP binding [49,105]. DSC can characterize the thermal denaturation of molecules and can provide information regarding the heat capacity of a solution as a function of temperature, by heating the reference and sample cells to the same temperature. It also can provide information regarding the heat changes during NP-PC formation, by observing the difference in the heat flow between the sample and reference cells as a function of time. Thus, the enthalpy changes of unfolding resulting from the heat-induced denaturation can be calculated, and the protein conformation after NP adsorption can be characterized.

The above approaches for characterizing NP-PC complexes are summarized in Table 1.

## 3. Applications in Biomedicine

The formation of a PC on the surface of an NP confers a new biological identity for the NP and offers myriad applications in biomedicines. The following subtopics will summarize recent developments in NP-PC systems for biomedical applications.

### 3.1. Regulating Cellular Uptake and Improving Drug Delivery

**Figure 8:**
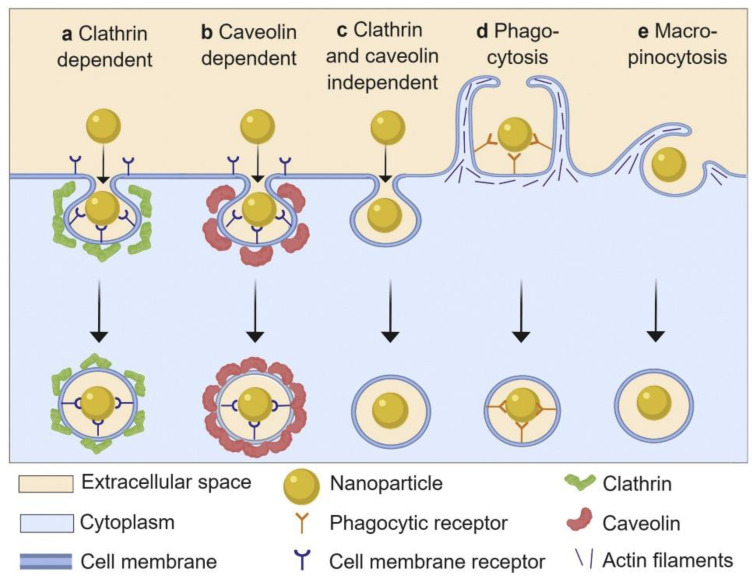
Schematic representation of basic nanoparticle uptake pathways. Reprinted with permission from [109]. Copyright (2019) Elsevier B.V.

The interactions of NPs with cells have gained increased attention in the context of biomedical applications, as the cell is a principal element for all organisms. Cell–NP interactions involve processes of recognition, uptake, and intracellular interactions. Herein, cellular uptake is one of the essential indicators for evaluating the potential of NPs for clinical applications. Cellular uptake can be described as follows: the cell membrane, which consists of lipid bilayers and membrane proteins, sinks partly and forms a vesicle to parcel the NPs inside; subsequently, the vesicle separates from the cell membrane and transports the NPs into the cells. As shown in Figure 8, the cellular uptake of NPs basically involves five pathways: phagocytosis, micropinocytosis, clathrin-dependent endocytosis, caveolin-dependent endocytosis, and clathrin- and caveolin-independent endocytosis [106,107,108,109]. It is reported that the cellular uptake of NPs is dependent on the size, shape, surface chemistry, and composition of the NPs [110,111,112,113]. While entering into cells, the NPs are instantly adsorbed with proteins. It is assumed that the interactions between the cells and NPs actually occur via interactions between cells and NP-PC complexes. The effects of PCs on the cellular uptake of NPs are manifold. PC formation on the surface of NPs can regulate cellular uptake by modulating the cellular response and uptake mechanism. In comparison with bare NPs, human serum PCs have been proven to improve the uptake of β-cyclodextrin-pluronic modified iron oxide magnetic NPs (MNPs) by human pancreatic carcinoma (Panc-1) cells and human bone metastatic LNCaP-derivative cancer cell line (C4-2B) prostate cancer cells [93]. It is reported that HSA coronas can improve the uptake of calcium carbonate nanocrystals in breast-cancer (MCF7), cervical-cancer (HeLa), and colon-carcinoma (Caco-2) cells [49]. In addition, the cellular uptake of gold nanorods (AuNRs) in Cal 27 oral squamous-cell carcinoma has been enhanced by an apolipoprotein E (ApoE) corona [114]. The improvement of their uptake by cancer cells reveals the enhanced bioavailability and biocompatibility of the above particles. It contributes to the effective loading of therapeutics in NPs in cancer cells. By contrast, the formation of fetal bovine serum (FBS) on the surface of ferroferric oxide NPs can minimize the nonspecific interactions between NPs and cells via lowering the NPs’ surface energy and inducing a lower cellular uptake efficiency in the NPs [115]. The cellular uptake of NPs on immune cells is decreased in the presence of a recombinant fusion protein [116] or immunoglobulin corona [117], which confers stealth properties on the NPs for evading immune cells and reduces clearance by macrophages. Correspondingly, it ensures the systematic targeting functions of the NPs, in vitro and in vivo. The above NP-PC complexes introduce potential for exploiting biomedical applications of NPs in drug delivery and cancer therapy.

In addition, the uptake of NPs might be either enhanced or inhibited by the adsorption of proteins, depending on the cell and/or protein types. AuNPs are widely used in drug delivery, cancer theranostics, diagnosis, and therapy, owing to their unique optical properties and good biocompatibility [118,119]. A series of studies by Monteiro-Riviere et al. revealed the effects of various PC coatings on the cellular uptake of AuNPs by different cells. It is reported that the formation of the HSA coronas on the surface of 80 nm polyethylene glycol (PEG)-AuNPs improves their uptake into human epidermal keratinocytes cells (HEK) [120], while reducing their uptake into human proximal tubule cells (HPTC) [121]. Upon coating 40 nm PEG-AuNPs with human plasma protein (HP), their uptake into hepatocytes increases [122], whereas the uptake into HEK [120] and HPTC [121] decreases. Similarly, while comparing the cellular uptake of lipoic acid (LA)-AuNPs or PEG-AuNPs with bare AuNPs, we can see that they are modulated by the pre-formed HP and HSA coronas, in opposite ways. That is, the cellular uptake of HP- or HSA-coated LA-AuNPs decreases, while the cellular uptake of HP- or HAS-coated PEG-AuNPs increases [122]. In addition to conventional AuNPs, the potential of PCs for the cellular uptake regulation of other NPs has also been exploited. It is reported that the pre-formed BSA corona on gelatin-oleic NPs can reduce their uptake in the human adenocarcinoma alveolar basal epithelial cells A549, while increasing their uptake in human embryonic kidney cells 293 [123]. Apolipoprotein 4 (ApoA4) or apolipoprotein C3 (ApoC3) coronas decrease the cellular uptake into the HeLa cells of NPs, while the pre-coating of NPs using apolipoprotein H (ApoH) enhances their cellular uptake [9]. Pre-formed ApoA4 or ApoC3 coronas can mask the nonspecific cellular uptake of NPs. Furthermore, the effects of different PCs on the cellular uptake of NPs have been studied using pre-coated amino-functionalized polystyrene NPs with human citrate plasma (HCP), human serum (HS), and human heparin plasma (HHP) [124]. The results show that the pre-coating of NPs using HCP induces more uptake into the HeLa cells than that via pre-coating using HS or HHP, whereas pre-coating using HHP induces a stronger uptake into the macrophage cell line than that via pre-coating using HS or HCP. The results reveal that the selection of an appropriate protein type for coating a given group of NPs is crucial for regulating their cellular uptake in specific cells.

**Figure 9:**
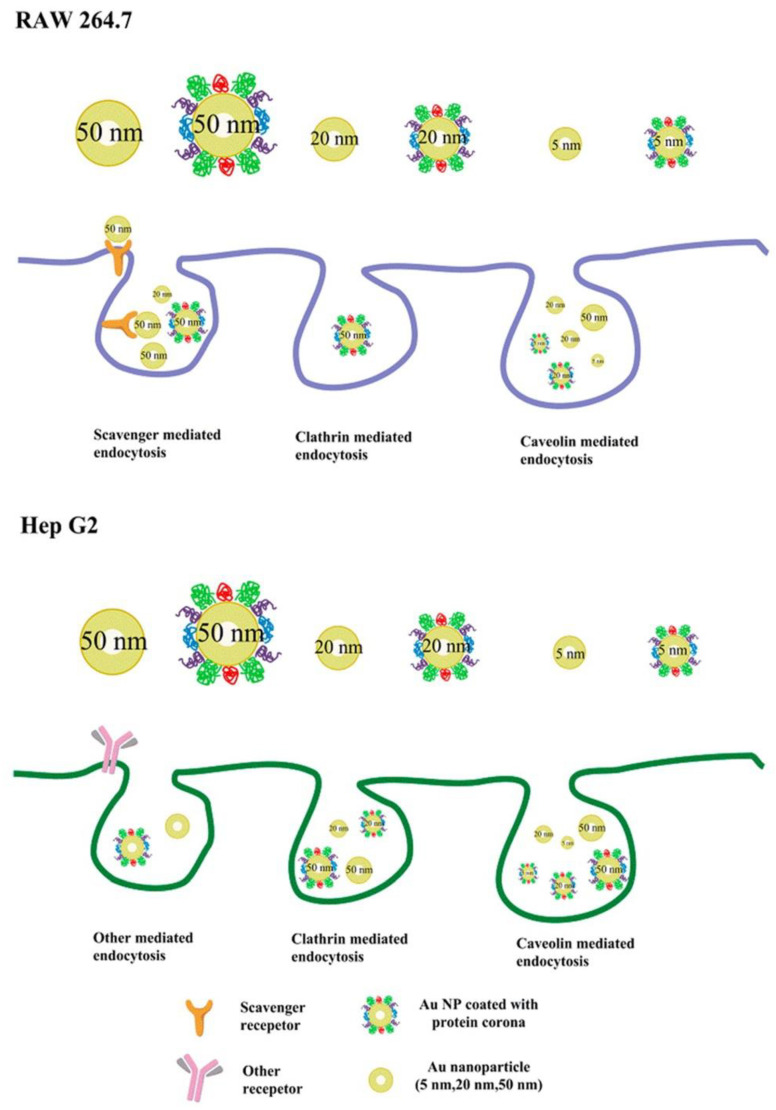
Proposed scheme of internalization pathways of differently sized AuNPs with or without PCs by RAW 264.7 and Hep G2 cells. Reprinted with permission from [25]. Copyright (2015) American Chemical Society.

Many studies have suggested that the effect of PCs on cellular uptake is also dependent on the size of the NPs. As differently sized AuNPs interact with cells via different endocytic pathways (Figure 9), FBS-coated 50 nm AuNPs show a significant inhibitory effect on the cellular uptake into the mouse leukemic monocyte macrophage cell line RAW 264.7 and human hepatocellular carcinoma cell line HepG2, whereas FBS-coated 20 or 2 nm AuNPs show almost no effect on the cellular uptake into both cell lines [25]. In the presence of a serum corona, silica NPs (SNPs) of 50, 100, and 200 nm diameters exhibit tremendous reductions in their uptake into M1 and M2 macrophages, in comparison with those in the absence of serum corona. However, the cellular uptake of 500 and 1000 nm SNPs by both types of cells shows slight changes in the presence and absence of serum corona [125]. Furthermore, significant increases in the uptake of 100 and 200 nm polystyrene NPs by human umbilical vein endothelial cells (HUVECs) have been observed in the presence of an FBS corona; however, almost no changes have been observed in the cellular uptake of FBS-coated polystyrene NPs (20 and 40 nm in diameter) [126]. It can be seen that the size of the NPs is an important factor affecting the interactions between NP-PC complexes and cells and should be considered while designing nanomedicines.

**Figure 10:**
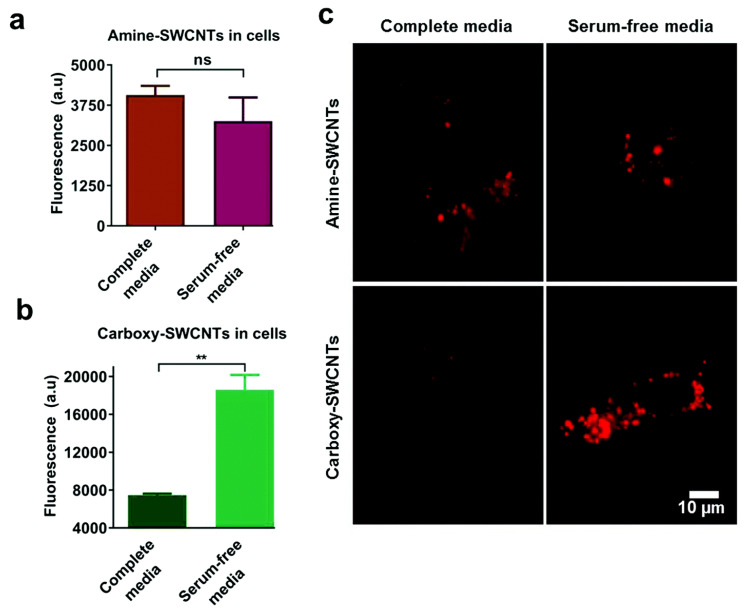
Effect of serum on uptake of polymer-single-walled carbon nanotubes (SWCNTs). Fluorescence emission from HeLa cell-associated (**a**) amine-SWCNTs and (**b**) carboxy-SWCNTs. (**c**) Representative near-infrared photoluminescence images of internalized SWCNTs in HeLa cells. Reprinted from [128] with permission from The Royal Society of Chemistry.

The surface chemistry of the NPs, including but not limited to the surface charge, hydrophobicity, and functional groups, also plays a key role in the cellular uptake process of NP-PC complexes. Among the factors regarding the surface chemistry, the surface charges of the NPs, owing to the interactions between the NPs and cell membranes or proteins in the serum, is a crucial factor. It is reported that mesoporous silica NPs (MSNs) with varying surface charges exhibit different protein adsorption capabilities. For MSN_c1 to MSN_c3, the amount of adsorbed serum proteins decreases with an increasing negative charge of the NPs, thus lowering the selective uptake of the MSNs by acute myeloid leukemia stem cells [127]. In addition to MSNs, in the presence of serum proteins, the cellular uptake of single-walled carbon nanotubes (SWCNTs) by HeLa cells is also highly dependent on their surface charges [128]. The serum protein coating induces drastic changes in the cellular uptake of anionic SWCNTs, whereas it has almost no effect on the cellular uptake of cationic SWCNTs (Figure 10). Furthermore, an impact of the surface chemistry of multiwalled carbon nanotubes (MWCNTs) on the uptake into macrophage RAW 264.7 cells has been reported [129]. The results show that a hydrophobic graphite sheet of pristine MWCNTs allows PCs to increase the particles’ water solubility and dispersibility and decrease their hydrodynamic size; thus, their uptake correspondingly increases. When MWCNTs are carboxylated, their sizes increase owing to protein adsorption, and they are less negatively charged, owing to the positively charged part of the proteins. All of these factors lead to a decrease in the cellular uptake of MWCNT-COOH. In addition to those regarding CNTs, the influences of the surface charges of AuNRs on the cellular uptake of NP-PC complexes have also been investigated. It has been shown that modifying AuNRs with the guest molecule pyr and host cage A confers a zwitterionic surface on the AuNRs and induces a reversible PC formation around the AuNRs, via host–guest interactions [130]. It increases the cellular internalization of the AuNRs into HeLa cells by up to 30-fold and regulates the cellular uptake of particles into phagocytic cells. In addition, it is noted that many stealth polymers have been employed in the decoration of NPs, thereby modulating PC formation. In comparison with non-decorated polystyrene NPs, less PC is coated on the surface of decorated stealth polymers, resulting in a reduction in macrophage cell line uptake [131]. Recently, the effect of the surface roughness on the cellular uptake of NPs has also been studied [132]. A differential uptake of NPs with different surface roughness values by the macrophage RAW 247.6 and MDA-MB-231 cell lines was found, as patchy NPs can noticeably reduce the adsorption of proteins, whereas a large amount of PC forms on spherical NPs with a smooth surface. The formation of serum PCs around NPs is generally considered to hinder the targeting efficiency of NPs. In the case of serum proteins coated with transferrin (Tf) functionalized virus-like particles, recent studies have indicated that they can successfully target cancer cells with transferrin receptors via the covalent coupling of Tf molecules on the capsid surface, i.e., the PCs have no effect on their uptake into tumor cells [133]. Tuning PCs by modulating the surface chemistry of NPs opens up great potential for the regulation of the non-specific cellular uptake of NP-PC complexes and for improving the therapeutic efficacy of nanomedicines. It is therefore important to consider multiple factors while applying surface modifications to modulate PC formation on NPs.

Appropriate PC coating around an NP could further improve the therapeutic loading capacity of the PC and consequently improve its therapeutic efficacy. Investigating the features that influence cellular uptake is an important task for fully understanding the cell–particle interactions, and for designing an efficient drug carrier. The above findings provide useful guidelines for the optimal design of future nanomaterials in drug delivery. More potentials of NP-PC complexes in cellular uptake should be exploited for the development of advanced nanomaterials.

### 3.2. Modulating Drug Release

Drug release refers to the process by which a drug loaded on NPs is released in the body, through the diffusion or dissolution of the NPs’ matrix, thereby releasing the drug in solution. Generally, the smaller the NPs, the larger the surface area-to-volume ratio, resulting in a faster drug release. By contrast, larger NPs allow for the loading of more drugs and exhibit a slower release. As NPs are subjected to physicochemical property changes in the presence of PCs, the manner by which the PCs affect the drug release of the NPs has become a crucial factor for evaluating promising nanomaterials for drugs and has also been a cause for concern.

The influence of PCs on drug release is inconsistent. It is reported that a BSA corona has the potential to stabilize the skeleton of poly-3-hydroxybutyrate-co-3-hydroxyhexanoate NPs and to confer a stealth effect to NPs [134]. It is promising for enhancing the biostability of drugs in liver homogenates and in organs. The BSA corona has also been shown to act as a physical barrier for limiting the permeation of coumarin-6 (Ce6) loaded on NPs into a medium, resulting in a significantly slower drug release than that from non-coronated NPs. By contrast, mouse serum-coronated AuNRs have been proven to enhance drug release, owing to their exchanges with plasma proteins in the blood [135]. These exchanges contribute to the high therapeutic efficacy in vivo and lead to complete tumor regression, without any discernible toxic side effects on the treated animals. We can see that endogenous serum-based PC-coated NPs have potential use in a diverse range of drug releases, instead of merely being treated as a hindrance. The appropriate choices of specific proteins to form PCs around NPs could thus further improve their therapeutic applications.

Proteins possess both hydrophobic and hydrophilic regions, and they can hold both of these types of drugs. The effects of protein-coated NPs on the release of different types of drugs have been investigated by loading the hydrophilic anti-cancer drug doxorubicin (Dox) and hydrophobic drug meloxicam (Mel) as well as their mixture on fetal calf serum (FeCS)-coated SNPs. The results demonstrate that the release of Dox is subject to significant enhancement during incubation at pH 4.8 as compared to that at pH 7.4, whereas the release of Mel shows no change. The differential drug releases at varying pH levels may be attributed to the increased water solubility of Dox at a lower pH via a proton-sensitive mechanism [136,137,138] and the stronger binding of Mel in the hydrophobic regions of the corona, as well as the low water solubility of Mel. Furthermore, the results show that the release and delivery of all loaded drugs are enhanced in the presence of a PC. Notably, coronated SNPs with mixtures of Dox and Mel loading exhibit the highest anti-proliferative efficiency. Such studies are promising for optimizing nanomedicine formulations and for enhancing the therapeutic efficiency of nanomaterials.

The modification of the surface chemistry of NPs is widely employed to enhance the biocompatibility of NPs for their drug delivery and therapeutic applications. Therefore, corona-based payload carriers with various surface functionalization have drawn increasing attention. In the presence of PCs, the release of Dox from magnetic mesoporous silica NPs (MMSNs) has been found to decrease from 84% to 41% over 24 h in comparison with that from free MMSNs, as the PCs cover the surfaces and pores of the porous particles and prevent the drug release from the NP cores [139]. However, when PEG is employed to functionalize the MMSNs, the protein adsorption is effectively reduced, inducing an increase in the Dox release from the NPs. In addition, PC-coated diversely charged NPs have been exploited to encapsulate Dox [140,141]. Positively charged Dox is loaded on HSA-coated AuNRs, which are functionalized using the cationic surfactant cetyltrimethylammonium bromide (CATB), anionic surfactant poly(styrenesulfonate), and non-ionic surfactant PEG. The results show that the HSA helps in impeding the burst release of Dox from the AuNRs. Among the three types of nanocarriers, functionalized CATB ones exhibit the largest amount of corona coating, and PEG functionalized ones exhibit the smallest amount of corona coating. Correspondingly, coronated CATB-AuNRs have the lowest drug release efficiency, whereas coronated PEG-AuNRs have the highest drug release efficiency. A similar modulation pattern for the release of Dox has been observed using FeCS-coated selenium NPs, which are functionalized using CATB, SDS, and the non-ionic surfactant Brij-58. This suggests that soft corona proteins such as those from HSA and FeCS can impede passive release and minimize the burst release effect for bound drugs in NPs. The reduced amount of corona tends to lead to the release of a higher payload. Coating different amounts of PC on the surface of NPs provides a promising approach to regulating the release of drugs from NPs. It will be conducive to the rational design of nanomedicines and help to overcome some of the limitations observed in the clinical translations of these nanotherapeutic systems.

### 3.3. Modulating Cytotoxicity and Immune Response

The interactions of cells with NPs have a significant effect on cellular behaviors and may induce cell death. The potential toxicity and risk of NPs in vivo have always been a great issue in the biomedical applications of nanomaterials. The PC confers a new intrinsic and biological identity to NPs, resulting in alterations in the interactions of NPs with cells and tissues, as well as a redefinition of the cytotoxicity and immunotoxicity of the NPs.

#### 3.3.1. Mitigating Bare NP-Mediated Cytotoxicity

The protective impacts of PC formation on bare NP-mediated cytotoxicity have been investigated in numerous studies [121,122,142,143]. Some of the toxic properties of NPs originate from the interactions between the NP surfaces and cell membranes. AgNPs are broadly used in various biomaterial applications, owing to their ideal/small size and potential antibacterial effects [144,145,146]. Their applications in the biological environment, however, have increased the risk of living organisms being exposed to AgNPs and have displayed detrimental impacts on human organs, including the cardiovascular system and central nervous system (CNS). A PC coating is influenced by physicochemical material properties and can affect the in vivo toxicity of NPs. It is reported that the addition of BSA, HSA, or high-density lipoprotein (HDL) is helpful in reducing the cellular uptake and cytotoxicity of AgNPs to rat lung epithelial (RLE) and rat aortic endothelial (RAEC) cells [147]. While entering into the body, AgNPs undergo dissolution and release Ag^+^ ions, owing to environmental changes [148,149]. The sustained release of Ag^+^ ions from the AgNPs, which may facilitate oxidative damage to adsorbed cells, is subject to a dose-dependent reduction in the presence of coronas. With an increasing dose of PCs, the surfaces of the AgNPs exposed to both cells decrease, and the AgNPs are stabilized by an increasing number of PCs, resulting in the reduced dissolution of the AgNPs. Correspondingly, the release of intracellular Ag^+^ is effectively inhibited, and AgNP-mediated toxicity is abated. In addition, a monomeric bilobed protein—bovine lactoferrin (BLf)—has been proven to ameliorate the AgNP-mediated cytotoxicity to human monocytic (THP-1) cells and to improve their bioavailability [150]. Similarly, coating BLf proteins increases the stability of the AgNPs and limits the rate of the Ag^+^ oxidative dissolution. It appropriately protects the cells from AgNP-mediated toxicity.

CNTs are another fascinating and advanced set of nanocarriers for drug delivery, owing to their unique mechanical, electrical, optical, and biological properties [151]. They possess a large surface area for drug loading and can cross mammalian cell membranes well [152]. However, it has been reported that CNTs may have adverse effects on cell proliferation and may induce oxidative damage, apoptosis, or necrosis in vitro [153,154,155]. With the increasing applications of CNTs, reducing CNT-mediated toxicity has become a crucial issue. CNTs may cause cumulative brain and nerve cell damage or CNS disorders, because they can enter the CNS via sensory nerves or by penetrating the blood–brain barrier via olfactory nerve pathways and the bloodstream. BSA has been proven to significantly reduce the cytotoxicity of CNTs in astrocytes, and the reduction effect is dependent on the size of the CNTs [156]. CNTs with a smaller diameter provide additional available surfaces for BSA binding. The BSA coronas formed in smaller CNTs are more compact and have more layers than those formed in larger CNTs. This leads to less surface exposure of the CNTs, increased cell viability, and reduced toxicity to cells. BSA coronas are also applied to mitigate the cytotoxicity of MWCNTs to HUVECs, via enhancing the internalization of particles and reducing oxidative stress [157]. Immunoglobulin G (IgG) has also been reported to lessen the MWCNT-mediated cytotoxicity to macrophages in RAW 264.7 cells [129]. The adsorption of BSA or IgG reduces the cellular uptake of the MWCNTs and prominently prevents cells from MWCNT damage, thus decreasing their cytotoxicity. Furthermore, it is reported that the formation of a PC in the plasma effectively reduces the proximity of SWCNTs to endothelial cells, thereby decreasing the cytotoxicity induced by the SWCNTs [158]. Notably, fibrinogen can rearrange on the surface of SWCNTs with the most number of layers and most compact form; hence, it displays more significantly attenuated effects regarding the toxicity of SWCNTs.

In addition, PCs have been demonstrated to reduce the cytotoxicity induced by other NPs. For example, the release of zinc ions from zinc oxide (ZnO) NPs can be tripped by the FBS corona, owing to the mutual electrostatic attraction with the protein, resulting in the inhibition of ZnO dissolution and lowering of its cytotoxicity [159]. The adsorption of the BSA corona can potentially weaken the associations between ZnO NPs and cells and decrease the cellular Zn elements, thereby reducing the cytotoxicity to THP-1 macrophages [160]. Similar results have been reported with nanodiamonds (NDs). Serum protein (HSA and BSA) coronas effectively shield the ND surfaces and mask the toxicity of the NDs to human lung epithelial cells [161].

#### 3.3.2. Enhancing Bare NP-Mediated Cytotoxicity

Ultrasmall AuNPs, with core sizes less than 3 nm, introduce great potential for exploiting new drug delivery mechanisms and have gained increasing attention [162,163,164]. Ultrasmall AuNPs have been proven to have little toxicity to BRL 3A rat liver cells; the HSA corona adsorbed on ultrasmall AuNPs undergoes a partial conformation transition from a β-sheet to an α-helix, thereby disrupting cell membranes and inducing cell apoptosis [165]. This indicates that HSA-coated AuNPs initially adhere to the cell membranes and are internalized by the cells. While penetrating into the cells, the corona AuNPs introduce nanoscale holes into the liver cells’ membranes and cause leakages of lactate dehydrogenase into the cell media, revealing the enhancing effect of the corona on AuNP-induced cytotoxicity. Despite the fact that AgNP-mediated cytotoxicity has been mitigated by many PCs as mentioned above, there are some coronas that play inverse roles. It is reported that serum protein-coated citrated AgNPs display higher cytotoxicity to mouse embryonic fibroblasts than that of non-coronated ones [166]. PC formation enhances internalization via the receptor-mediated endocytosis and cytotoxicity of citrated AgNPs via the mitochondrial pathway, whereas oligo(ethylene glycol)-alkanethiol (EG_6_OH)-coated AgNPs are more resistant to the adsorption of proteins, resulting in slight changes in their cytotoxicity. It is known that cells exposed to AgNPs undergo endoplasmic reticulum (ER) stress, leading to cellular apoptosis and toxicity. AgNPs coated with BSA, HDL, and FBS have been demonstrated to be transported into the ER, modifying the protein structure in the organelle and resulting in an enhanced ER stress response in comparison to pristine AgNPs [167]. An enhancement of cytotoxicity has also been found for FBS-coated cadmium sulfide nanomaterials (Cds NMs). FC-gamma receptor II (FcγRIIB) receptors’ expression is related to the protective functions of the immune system and has been reported to cause severe damage in humans. A pre-formed FBS corona on the surfaces of Cds NMs increases the abundance of proteins in the corona and enhances the expression of FcγRIIB receptors on the surface of rat lung macrophage cells [168]. The interactions between the receptors and proteins trigger cell apoptosis via the AKT/Caspase 3 signaling pathway and hence reinforce the cytotoxicity of the AgNPs.

#### 3.3.3. Regulating Immune Response

Injecting NPs into the body triggers processes and mechanisms that protect the host organism against foreign agents, as they are generally recognized as potential pathogens (e.g., viruses and bacteria). NPs can therefore provoke inflammatory and oxidative stress, which can ultimately result in cell death. Recent studies reveal that PC formation can alter the immunological identities of NPs [120,169,170,171]. It has been reported that black phosphorus (BP) materials can induce immunotoxicity and immune perturbation in macrophages in the presence of a plasma corona [172]. The adsorption of the PC activates the nuclear factor kappa-light-chain-enhancer of activated B cells (NF-kB) pathway and increases inflammatory cytokine secretion, suggesting a pro-inflammatory effect from PC-coated BP materials. The corona composition-dependent pro-inflammatory effect has been investigated for serum-coronated AgNPs in the context of THP-1 cells [173]. It has been suggested that BSA, HSA, and/or heat-inactivated FeCS coronas enhance the immune responses of THP-1 cells to AgNPs, and that the use of BSA and HSA stimulates different inflammatory cytokine secretions and higher activation in cells exposed to AgNPs, i.e., higher in 10% of heat inactivated FeCS than in 10% of HSA. In addition, BLf has been demonstrated to enhance the humoral immune response many-fold against the Bacillus anthracis protective antigen [150]. The addition of BSA and HSA coronas has been proven to decrease the inflammatory response in RLE and RAEC cells, whereas the HDL corona has been proven to enhance the inflammatory response in RLE cells [147]. In addition to AgNPs, PCs (BSA coronas) have been reported to enhance the inflammatory response to MWCNTs by inducing interleukin 6 (IL-6) and tumor necrosis factor (TNFα) release, as well as by enhancing the THP-1 monocytes’ adhesion to HUVECs [157].

The concept of PC adsorption contributes to new strategies for reducing potential nanohazards and for using a more rational design for nanoplatforms. It also is an important pathway for nanomaterials to stimulate an inflammatory response. The new biological identities are anticipated to improve the efficiency and bioavailability of NPs as potential drug delivery agents.

### 3.4. Regulating Protein Fibrillation

**Figure 11:**
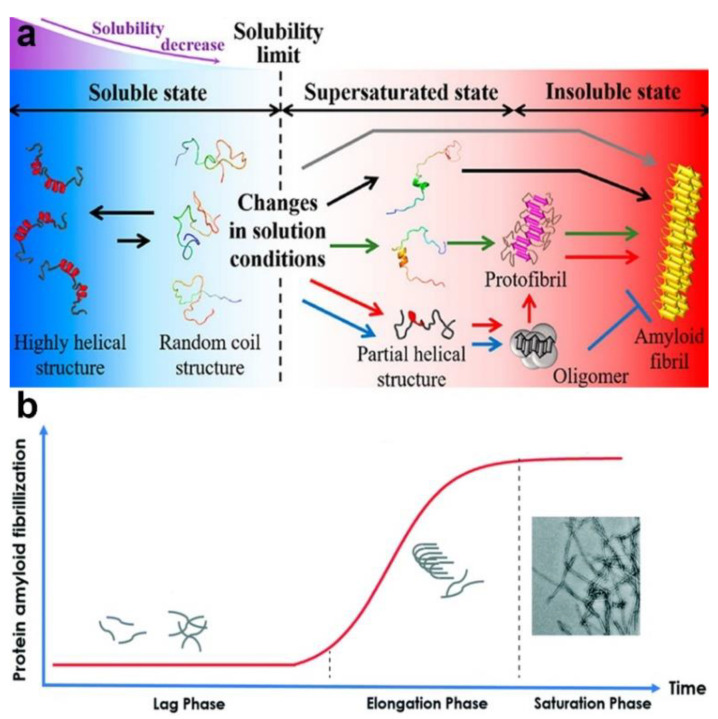
(**a**) Schematic representation of the protein fibrillation process. Reprinted with permission from [180]. Copyright (2019) American Chemical Society. (**b**) The kinetic process of protein amyloid fibrillization. Reproduced by permission from The Royal Society of Chemistry [181] with minor modification.

Protein fibrillation is a process by which soluble monomeric proteins self-assemble into largely insoluble amyloid fibrils characterized by a cross-β structure (Figure 11a). Basically, the kinetics of amyloidogenesis can be divided into three phases: a lag phase, elongation phase, and saturation phase (Figure 11b). Amyloidogenesis triggers a loss of the protein’s biological function and is involved in many neurodegenerative disorders such as Alzheimer’s disease (AD), Parkinson’s disease, and Huntington’s disease. The detailed mechanisms behind the fibrillation process remain elusive, and there is still a lack of specific therapeutic methods for protein fibrillation-related diseases. Researchers are still facing challenges in exploring the mechanisms of fibrillation formation and in exploiting effective approaches to inhibiting fibrillation in the human body. The development of NPs in biomedical applications offers a chance to address the current dilemma, as NPs have been suggested to inhibit the fibrillation process to a certain degree [174,175,176,177,178,179]. Correspondingly, for potential medicine materials, the key issues for investigation include the following: the instance when the NPs enter into the human body and how PC adsorption affects the inhibitory impacts of the NPs on protein fibrillation.

Amyloid beta (Aβ) fibrils are found in patients with AD. The amino acid sequences of 17–24, 30–36, and 38–42 are known as the hot spot regions of the Aβ peptide and have been widely identified as trigger factors of the fibrillation process. Bare AuNPs have been demonstrated to decrease the rate of the fibrillation process of Aβ_1–42_, as the self-assembly of the Aβ_1–42_ is impeded by the adsorption of the hot spot region 17–24 to the positively charged AuNPs [182]. The inhibitory effects of bare NPs have also been proven to be dose dependent. That is, a higher concentration of NPs offers a larger accessible surface for trapping higher amounts of Aβ_1–42_ monomers than a lower concentration of NPs, resulting in increased inhibitory effects. Corona-coated NPs show less inhibitory effects on fibrillation. PCs confer a negatively charged surface on the NPs, and the negatively charged parts of the hot spot region (17–24) display less binding affinity to corona-coated NPs, resulting in a reduction of the surfaces of the NPs exposed to the Aβ monomers. Moreover, corona-coated NPs have less capacity to trap Aβ monomers, facilitating the self-assembly of a large amount of Aβ monomers remaining in solution. In addition, the inhibitory effects of corona-coated NPs have been proven to be protein source- and concentration-dependent, owing to differences in the clotting factors of proteins. It shows that an FBS corona confers a larger inhibitory effect on NPs regarding Aβ fibrillation than that of an HP corona, whereas coronated NPs from 100% HP/FBS show less inhibitory effects than those from 10% HP/FBS.

**Figure 12:**
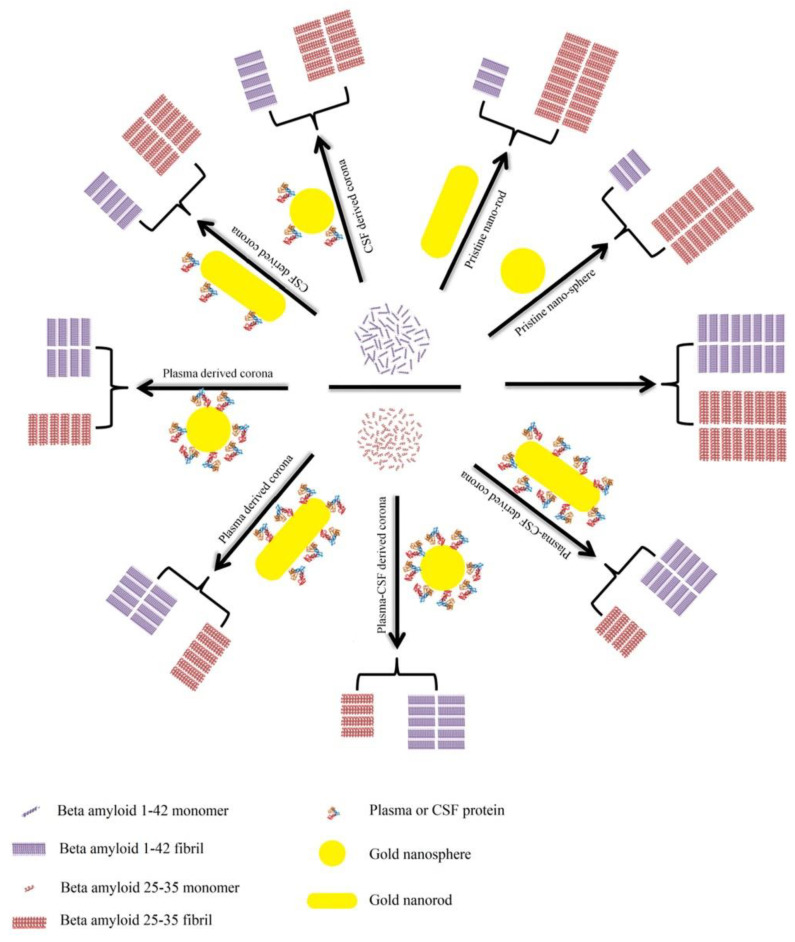
Schematic representation of the effects of bare/corona-coated gold nanospheres/nanorods on the fibrillation of Aβ_25–35_ and Aβ_1–42_. Reprinted with permission from [183]. Copyright (2018) American Chemical Society.

A detailed investigation has been conducted on Aβ_1–42_ and Aβ_25–35_ with human plasma or cerebrospinal fluid (CSF) coronas [183]. The inhibitory impacts are suggested to be based on the Aβ peptide and PC source (Figure 12). Generally, owing to the high capacity for capturing Aβ_1–42_ monomers, bare NPs demonstrate significant inhibitory effects on the Aβ_1–42_ fibrillation process. Compared with plasma-coated NPs, the surfaces of NPs, however, are not fully covered by the CSF corona, and hence, CSF-coated NPs possess more accessible surfaces for capturing Aβ_1–42_ monomers and for blocking the aggregation of hot spot regions. Therefore, plasma-coated AuNPs and AuNRs display less of an inhibitory impact on the Aβ_1–42_ fibrillation kinetics than CSF-coated or pristine AuNPs or AuNRs. In terms of the Aβ_25–35_ peptide, pristine NPs accelerate its fibrillation process, whereas corona-coated NPs inhibit the fibrillation process. There are several hydrophilic residues located in the N-terminal (25–30) of Aβ_25–35_, and several hydrophobic amino acids are located in the hot spot region (30–35). The hydrophilic NPs tend to bind to the hydrophilic region rather than the hot spot region; therefore, Aβ_25–35_ monomers concentrate on the NPs’ surfaces and tend to form fibrils driven by the unbound hot spot region.

In addition to Aβ peptide, an impact from the NP-PC complex on the fibrillation process of human islet amyloid polypeptide (IAPP), the fibrillation of which is a hallmark of type 2 diabetes, has also been reported [184]. β-lactoglobulin (bLg) is a typical β-sheet rich protein; its structure mostly turns into an α-helix after heat treatment. Introducing bLg and heat-denatured bLg-coated AuNPs to IAPP proves that the intercalation of bLg-AuNPs with IAPP is driven by β-sheet stacking. The fibrillation process of IAPP can be prolonged by bLg-coated AuNPs, as the interactions between the bLg-AuNPs and IAPP effectively block the IAPP self-assembly. It provides new clues for protein fibrillation detection and inhibition.

As there are limited studies regarding NP-PC complex-mediated protein fibrillation, it remains a significant challenge in clinical applications, and the current evidence on the inhibitory impacts of protein fibrillation has shown the valuable therapeutic potential of NPs for neurodegenerative diseases. NP-PC complexes are anticipated to advance our understanding regarding protein fibrillation and help in the design of NPs for biomedical applications. They provide new insights for overcoming challenges and into the limitations associated with traditional therapeutic methods and offer new hope to patients.

## 4. Conclusions

When NPs are introduced into a biological milieu, the protein-adsorbed NPs, rather than the bare NPs, represent the true identities and therapeutic responses of the NPs. Therefore, a comprehensive understanding regarding NP-PC complexes is very important for the development of new nanomaterials and for improving their availability in biomedicine and therapeutics. In this review, approaches for characterizing NP-PC complexes, including those based on the sizes, shapes, and surface charges of the NP-PC complexes, or the binding capacities, compositions, and/or conformational changes of PCs have been evaluated. Furthermore, recent biomedical applications of NP-PC complexes have been summarized. Although several attempts have been made to characterize NP-PC complexes and the applications of NP-PC in biomedicines and have shown a remarkable increase, it is too early to say that we have a clear understanding for appropriate applications regarding NP-PC complexes. Multi-disciplinary approaches are needed to obtain more information regarding PCs and their impacts in nanomedicines to achieve the desired biological and therapeutic outcomes with nanomaterials. Thus, this study anticipates improved support for safer and more conscious applications of nanotechnology in medicine.

## Figures and Tables

**Table 1:** Approaches for characterizing nanoparticle–protein corona (NP-PC) complexes.

Characterization	Approach	Brief Description
Size, shape, and surface charge	Dynamic light scattering (DLS)	Size distribution and hydrodynamic sizes
	Zeta-potential	Surface charge
	Differential centrifugal sedimentation (DCS)	Size analysis
	Transmission electron microscopy (TEM) or atomic force microscopy (AFM)	Morphology of NPs and thickness of PCs
	Small-angle X-ray scattering (SAXS)	Size and shape of particles
	NP tracking analysis (NTA)	Particle concentration and size
Binding capacity	UV-vis	Changes in light absorption spectra
	Fluorescence correlation spectroscopy (FCS)	Changes in diffusion time of fluorescently labelled particles
	Quartz crystal microbalancing (QCM)	Mass changes at the oscillating quartz surface
	Isothermal titration calorimetry (ITC)	Thermal changes induced by NP-PC binding
	Nuclear magnetic resonance (NMR)	Binding site location
	Computer simulation	Protein orientation and conformation
PC composition	Gel electrophoresis	Molecular weight estimation
	Mass spectrometry (MS)	Identification of each protein component
	Gel filtration chromatography	Protein separation and kinetic exchange rate calculation
PC conformation	Circular dichroism (CD)	Secondary structural changes
	Surface-enhanced Raman spectroscopy (SERS)	Tracks molecular vibrations for protein conformation analysis
	Fourier-transform infrared spectroscopy (FTIR)	Changes in absorption of infrared light
